# Mechanical Vibration Influences the Perception of Electrovibration

**DOI:** 10.1038/s41598-018-22865-x

**Published:** 2018-03-14

**Authors:** Semin Ryu, Dongbum Pyo, Soo-Chul Lim, Dong-Soo Kwon

**Affiliations:** 1 Institute for Integrated Studies, Hallym University, 1 Hallymdaehak-gil, Chuncheon-si, Gangwon-do 24252 Republic of Korea; 2 Electronics and Telecommunications Research Institute (ETRI), 218 Gajeong-ro, Yuseong-gu, Daejeon 34129 Republic of Korea; 3 Department of Mechanical, Robotics and Energy Engineering, Dongguk University-Seoul, 30 Pildong-ro 1-gil, Jung-gu, Seoul 04620 Republic of Korea; 4 Department of Mechanical Engineering, Korea Advanced Institute of Science and Technology (KAIST), 291 Daehak-ro, Yuseong-gu, Daejeon 34141 Republic of Korea

## Abstract

Recently, various methods using, simultaneously, two types of tactile feedback have been proposed to emulate a real object. However, the possible masking effect when providing two types of tactile feedback has been scarcely reported. In this study, we investigated the masking effect caused by mechanical vibration on the perception of electrovibration. The absolute and difference thresholds of the electrovibration were measured according to the presence/absence, frequency, and intensity of the mechanical vibration. The absolute threshold of electrovibration tended to increase in the form of a ramp function, as the intensity of the masking stimulus (mechanical vibration) increased. Particularly, the masking effect was more remarkable when the frequency of both the target and the masking stimulus was the same (up to 13 dB increase with 25 dB SL masker). Furthermore, the difference in the threshold (average of 1.21 dB) did not significantly change due to the masking stimulus, when the sensation level intensity of the target stimulus was within the section following the Weber’s law. The results further indicated that electrovibration contributes to the activation of slowly adapting afferents as well. This investigation will provide important guidelines for the design of haptic interface that employs multiple types of tactile feedback.

## Introduction

The importance of haptic feedback has been recognized in a diversity of fields, from the design of tactile devices to virtual reality^1^. More specifically, haptic interaction on touch surfaces has attracted great attention with the diffusion of touch screen-based devices. Research has actively been conducted in various fields, and includes the development of actuators and sensors^2,3^, psychophysical studies^4,5^, and rendering techniques^6,7^, in order to convey meaningful tactile information on touch surfaces. To provide haptic feedback on a touch surface, a method for generating mechanical vibration using a vibrotactile actuator has been widely used. Theories and methods for simulating surface texture by transmitting vibrations to the touch surface or to a tool, such as the stylus, have been studied, and mechanical vibrations have proven to be one of the most effective methods for reproducing such textures^2,4,5,7,8^. As another means of providing haptic feedback on a touch surface, friction modulation methods that are based on the squeeze film effect or the electrovibration principle have been investigated^9–13^ as well. More specifically, electrovibration has been intensively studied and commercialized^14^ owing to its potential for seamless integration into ordinary touch screen-based devices. Electrovibration can increase the dynamic friction that is perceived by users based on the electrostatic force that is induced between the sliding fingertip and the touch surface^11^. By modulating the perceived friction, the electrovibration could simulate various surface textures or 3D shapes of real objects on a touch surface^6,15,16^. The usefulness of such haptic feedback has recently led to the development of vibrotactile actuators and electrovibration displays that have a thin and flexible form; these are directed toward future wearable electronics^3,17–19^.

As previously mentioned, among the various types of tactile information, methods for reproducing the *shape* or *texture* of objects on a touch surface have been mainly considered. However, most studies have focused on reproducing a single piece of information (either shape *or* texture) with a single tactile feedback (either vibration *or* friction). To emulate haptic responses that are similar to those when touching a real object, it is desirable to stimulate multiple tactile factors simultaneously^20^. In other words, it may be effective to jointly use multiple types of tactile feedback. In this regard, certain research groups have proposed manners in which two types of tactile feedback (vibration + friction or lateral force) may be used simultaneously^21–23^. By using two types of tactile feedback, they demonstrated the feasibility of the simultaneous representation of the shape and texture and/or key-click feeling and texture on a touch surface. Nevertheless, relatively little consideration was given to the perceptual characteristics of humans when the two types of tactile feedback were mixed. In general, when two or more stimuli are provided together, a masking effect (the perception of one stimulus is affected by the presence of another stimulus) acts on the perception of the stimulus. The masking effect appears in the visual^24^ and auditory senses^25,26^, as well as in the tactile sense^27,28^, even in different sensory modalities^29^. The masking effect is typically examined by observing changes in the absolute thresholds (absolute limen, AL) and the difference thresholds (difference limen, DL) of the target stimulus, depending on the presence or absence of the masking stimulus^30^. In tactile perception, the masking effect has been investigated in various manners by varying the location, frequency, intensity, stimulus-onset asynchrony, etc. of the target and the masking stimulus^27,28,31,32^. It was observed that the absolute threshold for the intensity of the stimulus tended to rise when there was a masking stimulus, in contrast to when no masking stimulus was present^33^. The masking effect on the absolute threshold was more prominent when the target stimulus and the masking stimulus were provided at the same site than when they were provided at different sites^31^, or when the same sensory channel was stimulated than when different sensory channels were stimulated^27^ (Pacinian and non-Pacinian channels). In addition, the absolute threshold increased as the frequencies of the target stimulus and the masking stimulus became similar to each other or when the intensity of the masking stimulus became stronger^27,33^. Although the tendency of changes in the absolute threshold was similar in several research works, the extent of changes in the absolute threshold owing to masking stimuli has been observed to be different, according to the conditions of the stimuli; this has been observed in the difference threshold experiments as well^28,30,34^. To date, most studies have been focused on the investigation of the masking effect when the target stimulus and the masking stimuli were provided through the same tactile feedback means. However, the masking effect when different types of tactile feedback are provided has been scarcely studied. As previously described, the masking effect has been observed to be different depending on the condition of the stimulus; therefore, it should be taken into account, particularly in the design of a haptic interface using a new combination of types of tactile feedback.

In this study, we considered simultaneously providing two entirely different tactile feedback means, namely electrovibration (EV) and mechanical vibration (MV), to the bare fingertip that would slide on the touch screen. More specifically, as a first step, the present study was focused on the perception of electrovibration in the presence of mechanical vibration. The main objective was to investigate the changes in the absolute and difference thresholds of the electrovibration when the mechanical vibration was provided as a masking stimulus. To measure these thresholds, we constructed the hardware setup in a manner that it would provide both electrovibration and mechanical vibration on the touch surface. We then conducted three experiments with a group of participants to confirm the effect of mechanical vibration on the perception of electrovibration. All experiments were conducted in a manner of active touch. First, in Experiment 1, we investigated the absolute threshold of each of the electrovibrations and mechanical vibrations. Then, in Experiment 2, we measured the masked absolute threshold of the electrovibration when the mechanical vibration with various frequencies and intensities was provided as the masking stimulus. The intensity of the masking stimulus was provided in units of dB SL (where SL is the sensation level; it refers to the number of decibels above the absolute threshold), and was calculated based on the absolute threshold of the mechanical vibration that had been revealed in Experiment 1. Finally, in Experiment 3, we measured the difference threshold of the electrovibration with the presence/absence of the masking stimulus. In this case, the electrovibration was provided at a constant intensity, the sensation level of which was based on the masked threshold that had been revealed in Experiment 2. The masking stimulus was the same as that employed in Experiment 2.

## Results

### Experiment 1

**Figure 1:**
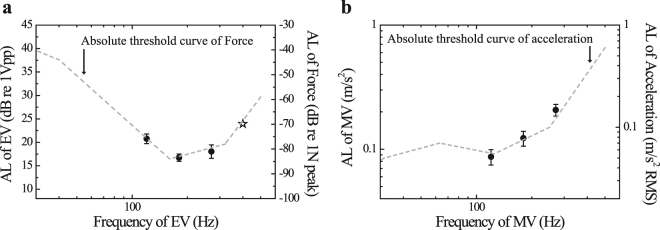
Absolute thresholds (AL) of electrovibration (EV) and mechanical vibration (MV). (**a**) Mean absolute threshold of electrovibration for three test frequencies with standard error bars (filled circle, left axis) and the absolute threshold curve of the tangential force, as obtained by Israr *et al*.^35^ (dashed line, right axis). The open-star symbol, which was not used for analysis, denotes the roughly estimated absolute threshold of electrovibration at 400 Hz, as obtained from a pilot test. (**b**) Mean absolute threshold of mechanical vibration for three test frequencies with standard error bars (filled circle, left axis) and the absolute threshold curve of acceleration, as obtained by Morioka *et al*.^36^ (dashed line, right axis).

Figure 1a shows the results from Experiment 1, namely the mean absolute threshold of the electrovibration for three test frequencies (filled circle) with standard error bars. The absolute threshold of the electrovibration is expressed in units of “dB re 1 Vpp”; it was calculated as 20 × log_10_(*A*), where *A* is the signal amplitude in volts (peak-to-peak), and is plotted on the left axis. Normality was assumed by performing the Shapiro–Wilk test (*p* > 0.05 for all frequencies). Mauchly’s test did not present a violation of sphericity against frequency (*W*_2_ = 0.914, *p* = 0.699). The effect of frequency on the absolute threshold levels was statistically significant; this was confirmed by a one-way repeated-measures analysis of variance (ANOVA) (*F*_2,18_ = 4.289, *p* < 0.05, and *η*_*p*_^2^ = 0.323), indicating that the threshold levels depend on the frequency. For comparison, the absolute threshold curve for a force acting in a tangential (lateral) direction with respect to the fingertip—obtained from the work of Israr *et al*.^35^—is plotted (dashed line) in Fig. 1a. In addition, the absolute threshold of the electrovibration at 400 Hz, which was roughly estimated through a pilot test, is displayed as well (open star). The absolute threshold of the electrovibration and the force (lateral) exhibits a similar behavior.

In addition, the absolute threshold of the mechanical vibration was measured in units of “dB re 1 Vpp”. The estimated mean absolute threshold levels were 10.538 ± 0.934, 5.593 ± 1.577, and 17.779 ± 0.991 dB (mean ± standard error) for the test frequencies of 120, 180, and 270 Hz, respectively. However, as the output characteristics of the mechanical vibration were frequency-dependent (see Supplementary Fig. S5), the aforementioned results were converted to acceleration (see Supplementary Fig. S6) for comparison purposes. Figure 1b shows the results from Experiment 1, namely the mean absolute threshold of mechanical vibration for three test frequencies (filled circle) with standard error bars, converted to acceleration. Normality and sphericity were assumed based on the results from the Shapiro–Wilk test (*p* > 0.05 for all frequencies) and Mauchly’s test (*W*_2_ = 0.631, *p* = 0.158). A one-way repeated-measures ANOVA revealed that the effect of frequency on the absolute threshold levels was statistically significant (*F*_2,18_ = 43.303, *p* < 0.001, and *η*_*p*_^2^ = 0.828), indicating that the threshold levels of mechanical vibration depend on the frequency as well. For comparison purposes, the absolute threshold curve of the mechanical vibration that acts vertically at the fingertip—as presented by Morioka *et al*.^36^ for passive touch—is plotted as well (dashed line in Fig. 1b); both threshold curves present similar trends.

### Experiment 2

**Figure 2:**
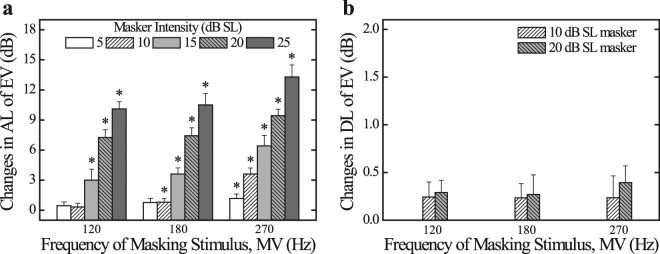
Changes in the absolute threshold (AL) and difference threshold (DL) of electrovibration (EV) in the presence of mechanical vibration (MV). (**a**) Mean changes in the absolute threshold of EV at 270 Hz under masker conditions (120, 180, and 270 Hz with 5–25 dB SL) with standard error bars. To evaluate the masking effects, the unmasked ALs of EV at 270 Hz in Experiment 1 were subtracted from the corresponding masked ALs, which were measured in Experiment 2. Asterisks denote that the data column is significantly different from zero (*p* < 0.0.05). (**b**) Mean changes in the difference threshold of EV at 270 Hz under masker conditions (120, 180, and 270 Hz with 10 dB SL and 20 dB SL) with standard error bars. To evaluate the masking effects, the unmasked DLs of EV at 270 Hz were subtracted from the corresponding masked DLs.

Figure 2a shows the average changes in the absolute threshold of the electrovibration at 270 Hz in the presence of mechanical vibration, along with the corresponding standard error bars. Normality and sphericity were checked based on the results of the Shapiro–Wilk test (*p* > 0.05 for all cases) and Mauchly’s test (*p* > 0.05 for frequency and interaction term, *p* < 0.05 for amplitude). A two-way repeated-measures ANOVA showed that the effects of both frequency and amplitude, on the changes in AL levels, were statistically significant (*F*_2,18_ = 5.960, *p* < 0.05, and *η*_*p*_^2^ = 0.398 for the frequency; *F*_1.69,15.22_ = 189.233, *p* < 0.001, and *η*_*p*_^2^ = 0.955 for the amplitude), whereas the effect of the interaction term was not statistically significant (*F*_8,72_ = 1.171, *p* = 0.328, and *η*_*p*_^2^ = 0.115). More specifically, the absolute threshold of electrovibration tended to increase as the intensity of the masking stimulus increased. Regarding the frequency, a multiple-paired *t*-test with a Bonferroni correction revealed that the changes in ALs were greater in the 270 Hz masker conditions than in the 120 Hz and 180 Hz masker conditions (*p* = 1.000 for the 120 Hz–180 Hz pair, *p* < 0.05 for the 120 Hz–270 Hz and the 180 Hz–270 Hz pairs). That is, the masking effect became more prominent when the frequencies of electrovibration and mechanical vibration were the same. To determine whether the masking effects were present or absent, two-tailed one-sample *t*-tests were performed on all data columns. The asterisk in Fig. 2a indicates that the corresponding data column is significantly different from zero (*p* < 0.05). When the frequency of the target stimulus (electrovibration) and those of the masking stimulus (mechanical vibration) were identical, a masking effect was observed in all data columns (5–25 dB SL masker conditions). Furthermore, when the frequencies of EV and MV are different, a masking effect was observed only when the intensity of the masking stimulus was equal to, or more than, 10 dB SL and 5 dB SL for the 120 Hz and 180 Hz masker conditions, respectively. It is noteworthy that the changes in the AL of the EV showed a linear relationship (slope of approximately 0.6 for 120/180 Hz maskers and 0.66 for 270 Hz masker, with an adjusted R-square > 0.0.99), after the absolute threshold of EV began to increase.

### Experiment 3

Figure 2b shows the average changes in the difference threshold of the electrovibration in the presence of mechanical vibration, along with the corresponding standard error bars. Normality and sphericity were assumed based on the results from the Shapiro–Wilk test (*p* > 0.05 for all cases) and Mauchly’s test (*p* > 0.05 for all effects, including the interaction term). A two-way repeated-measures ANOVA failed to show the significant effects of the frequency, amplitude, and interaction term on the difference threshold levels (*F*_2,18_ = 0.085, *p* = 0.919, and *η*_*p*_^2^ = 0.009 for the frequency; *F*_1,9_ = 0.708, *p* = 0.422, and *η*_*p*_^2^ = 0.073 for the amplitude; *F*_2,18_ = 0.129, *p* = 0.880, and *η*_*p*_^2^ = 0.014 for the interaction term). In other words, the amount of changes in DLs of EV did not significantly vary as a function of the frequency and intensity of the masking stimulus (MV). To identify whether masking effects were present or absent, two-tailed one-sample *t*-tests were performed on all data columns. The tests showed that all data columns were not significantly different from zero (*p* > 0.05 for all cases), indicating that despite the existence of a masking stimulus (MV), the difference threshold of EV does not significantly change if the target stimulus (EV) is equated at the sensation level. The average difference threshold for all test sets was 1.21 dB.

## Discussion

For the perpendicular stimulus to the skin, the presence or the frequency responses of different mechanoreceptive fibers (or sensory channels) have been revealed by investigating the masking effect on the absolute thresholds using stimuli that stimulate different afferents^37,38^. Moreover, the response of a sensory channel to the tangential (or lateral) stimulus to the skin still has not been completely understood, despite many researchers have investigated the tangential stimulus^39–41^. More specifically, the perception of electrovibration has been rarely reported so far, while a number of studies have focused on the physics of the EV and the influence of the waveform^42–45^. In this study, we investigated the masking effects that were caused by mechanical vibration when the electrovibration was perceived. First, in Experiment 1, the absolute threshold of the electrovibration and the mechanical vibration in the active-touch mode was measured. In the present study, the measured absolute thresholds of mechanical vibration were used as a reference value to obtain the intensity of the masking stimulus at the same sensation level. In Experiment 2, there were more masking effects when the frequency of the masking stimulus (MV) was equal to that of the target stimulus (EV), or as the intensity of the masking stimulus increased. On the other hand, in Experiment 3, it was confirmed that the difference threshold of electrovibration did not significantly change with the presence or absence of the masking stimulus.

The electrostatic attractive force that is induced by electrovibration acts as friction^13,15^ on the moving fingers; however, it does not give any feedback to stationary fingers^11^. That is, electrovibration is the feedback that is provided in the direction that is lateral (or tangential) to the skin. Therefore, there is a similar tendency for the frequency when compared with the threshold curve of the force that acts in the tangential direction (see Fig. 1b), indicating that humans perceive the electrovibration in a similar manner to the lateral force. Although studies on thresholds of mechanical vibration have been conducted on various body parts, they have been mainly limited to passive touch; research on active touch is scarce. According to Ryu *et al*.^46^ and Morioka and Griffin^36^, the absolute threshold of the mechanical vibration that is provided to the hand through passive touch was the lowest at approximately 120 Hz–160 Hz, and then tended to increase with the increase in frequency. In the present study, the absolute threshold of the mechanical vibration that is provided through active touch tended to increase after 120 Hz. In general, the level of the absolute threshold depends on the location and the contact area of the stimulus^36,47^. Therefore, it is difficult to discuss the manner in which the threshold level changes depending on the touch mode (passive or active) by considering only the results of the present study. However, as shown in Fig. 1b, the tendency of two absolute threshold curves as a function of the frequency was similar, so it can be assumed that there is no significant change in frequency-dependent characteristics that may be attributed to active or passive touch when a human perceives a mechanical vibration.

In general, the masking effect on the absolute thresholds became greater when two stimuli activated the same sensory channels than when the stimuli activated different sensory channels (e.g., Pacinian and non-Pacinian channels)^27,31,33^. In particular, even within the same sensory channel, the change in the absolute threshold was greater when a masking stimulus, at a frequency similar to that of the target stimulus, was provided^33^. In addition, in both temporal and simultaneous masking, the absolute threshold tended to increase as the intensity of the masking stimulus increased^33,48^. The masked absolute thresholds of electrovibration, which were investigated in this study, were found to vary significantly when the intensity of the masking stimulus (MV) was greater, or when the frequencies of the target stimulus (EV) and the masking stimulus were the same. When the intensity of the masking stimulus was 10 dB SL, the absolute threshold of the electrovibration did not significantly change for masking stimuli at different frequencies, and it increased by approximately 3 dB for the masking stimulus of the same frequency. When the intensity of the masking stimulus was 20 dB SL, the absolute threshold of the electrovibration increased by approximately 6.5 dB and 10 dB for the masking stimuli of the same and different frequencies, see Fig. 2a. In the works of Verrillo *et al*.^31^ and Hamer *et al*.^27^, the changes in the absolute threshold ranged from approximately 0 to 3 dB and 7 to 10 dB for 10 dB SL and 20 dB SL masker conditions, respectively, when the target and masking stimuli with the same frequency (200 Hz or 300 Hz) were introduced to the thenar eminence or the fingertip. In the present study, the changes in the absolute threshold of the EV obtained from the 270 Hz masker conditions, 3.6 dB and 9.4 dB for the 10 dB SL and 20 dB SL masker conditions, respectively, were similar to those reported in previous studies. The tendency of the masking effect to become more prominent when the frequencies of the target and the masking stimulus were the same (see Fig. 2a) was consistent with that reported by Verrillo^33^. In previous studies by other research groups, the same type of tactile feedback (mechanical vibration only) was used as a target and masking stimulus in a passive-touch manner^27,31,33^. Furthermore, in the present study, different types of tactile feedback (electrovibration and mechanical vibration) were provided in an active-touch manner. It was found that the masking effect presented a similar tendency for different touch modes and types of stimuli.

The masking function of electrovibration was observed in the form of a ramp function. After the absolute threshold began to increase, the masking function was almost linear, having a slope of approximately 0.6–0.7. This result is consistent with the result of sinusoidal cross-channel masking experiment reported by Hamer *et al*.^27^ and Gescheider *et al*.^37^. In another aspect, it is remarkable that there is a region in which the masking effect does not appear (i.e., the amount of change in the absolute threshold is not significantly different from zero). As revealed by Hammer *et al*.^27^, this phenomenon has been observed when the different sensory channels are activated by the target and the masking stimulus. Since the rapidly adapting type 2 (RA II) afferents are most sensitive at frequencies of 120–270 Hz, for stimuli acting perpendicular to the skin, the mechanical vibration used in the current study would contribute mainly to the activation of RA II afferents. Thus, from the results in Fig. 2a, electrovibration can be interpreted as activating at least one of the slow adapting (SA) afferents. In other words, the existence of the region where the masking effect does not appear would be evidence that the SA afferents respond sensitively to the electrovibration. More specifically, the authors expect that the SA II afferents would respond to the electrovibration since they are 2–4 times more sensitive to skin stretch than the SA I afferents^49,50^. We also paid attention to the perception of the mechanical vibration. According to the results of the cross-channel masking effect by Hamer *et al*.^27^, the masking effect was observed when the intensity of the masking stimulus was sufficiently increased in order to activate the same sensory channel as the target stimulus. Typically, the activation threshold of SA afferents for mechanical vibration (vertical direction with passive-touch mode) has been known to be significantly higher than that of RA afferents (more than 20–30 dB for the frequencies above 100 Hz)^51^. However, Fig. 2a shows that the masking effect begins to appear when the intensity of the masking stimulus is equal to, or less than, approximately 10 dB SL. Thus, it could be deduced that the difference in the activation threshold between RA and SA afferents, for mechanical vibration with active-touch mode, is less than 10 dB at the frequency range of 120–180 Hz. In particular, the masking effect appeared regardless of the intensity of the masking stimulus when the frequency of MV was 270 Hz. Thus, the activation threshold of SA afferents, for MV provided in an active touch manner, would be equal to, or lower than, that of RA afferents at that frequency. As described so far, the masking effect is clearly present in a combination of different types of stimuli (EV + MV) as well. Therefore, it is essential to consider the masking effect in the design of a tactile feedback when two or more stimuli are combined.

The average difference threshold for all test sets was 1.21 dB, which was also consistent with the results obtained by Bau *et al*.^11^ (1.16 dB for electrovibration only). The presence/absence, frequency, and intensity of the masking stimulus (MV) did not cause any significant change in the difference threshold of electrovibration in the 15 dB SL condition. On the other hand, Israr and Tan^30^ revealed that the average difference threshold of the target stimulus increased by approximately 3.7 dB when a masking stimulus was present. This is contradictory to the results of the present study because Israr and Tan^30^ determined the intensity of the target stimulus at the sensation level based on the nominal threshold (unmasked threshold). In general, the amplitude difference threshold of vibration is known to be constant when the intensity of the target stimulus is approximately 10 to 15 dB SL or more, that is, it follows Weber’s law^52–54^. When determining the intensity of the target stimulus based on the unmasked threshold, the target stimulus may not be perceived at a constant sensation level in the presence of various types of masking stimuli. The difference threshold would increase if the masking stimulus causes the perceived intensity of the target stimulus to decrease to less than 10–15 dB SL by. In the present study, the intensity of the target stimulus (EV) was constantly provided at 15 dB above the masked threshold that was examined in Experiment 2. As a result, the masking stimulus did not cause significant changes in the difference threshold of electrovibration, which is consistent with the results from other studies in which the masked threshold was considered^28,34^. Therefore, even when two different types of tactile feedback (EV + MV) are simultaneously provided, a feedback design that assumes the difference threshold to be constant would become possible, especially, if the intensity of the target stimulus is determined based on the masked threshold and is within the section of the psychometric curve following the Weber’s law.

## Conclusion

The absolute and difference thresholds are the most basic and important factors in the design of a haptic feedback. However, if there is a masking effect, a change may certainly occur in the absolute and/or difference threshold. Even with a combination of two stimuli with completely different mechanisms such as the combination of electrovibration and mechanical vibration, a significant masking effect would be observed. In the present study, we investigated the absolute and difference thresholds of the electrovibration when a mechanical vibration was provided as a masking stimulus. As a result, the absolute threshold of the electrovibration has a masking function in the form of a ramp function. Furthermore the difference threshold of the electrovibration does not significantly change, irrespective of the masking stimulus in the interval following Weber’s law. In practical terms, the results of the present study provide important guidelines for the design of haptic interfaces that use two stimuli in conjunction. There are quite a few examples of research that have suggested using two stimuli of different mechanisms simultaneously for haptic interface design^21–23,55^. For the design of a haptic interface using EV and MV^21,55^, we suggest considering the changes in AL as a ramp function, as shown in Fig. 2a. In order to provide an EV, to be perceived at a specific intensity in a certain sensation level, it is desirable to provide the EV with a stronger intensity considering the masking function. In addition, for the stimulus intensity following the Weber’s law, designers would not need to consider the difference threshold of the EV as a function of the masking stimulus. The authors would also recommend researchers to apply the approach described in the current study, not only in the case of using EV and MV, but also in other combinations such as using active force and vibration in conjunction^22,23^. The current study was focused on the influence of the masking effect on the perception of electrovibration, that is, the masking effect on one of the two stimuli. However, masking effects may certainly appear in the perception of a mechanical vibration. In this case, the results might be somewhat different from the results of the present study. In addition, designing haptic feedback that can independently provide two stimuli at the desired intensities by considering ‘mutual’ masking effect may be a more complex problem. These issues will be discussed in future studies.

## Methods

### Participants

In total, ten participants took part in the experiment. Nine participants were males and one was female. Their age was 26.1 ± 2.73 years (mean ± standard deviation). All were right-handed, and no known cutaneous or kinesthetic sensing problems were reported. Prior to the experiments, they received written and verbal instructions, and gave informed consent. All experiments were approved by the Institutional Review Board (IRB) of the Korea Advanced Institute of Science and Technology (KAIST), and were conducted in accordance with the approved guidelines.

### Apparatus

**Figure 3:**
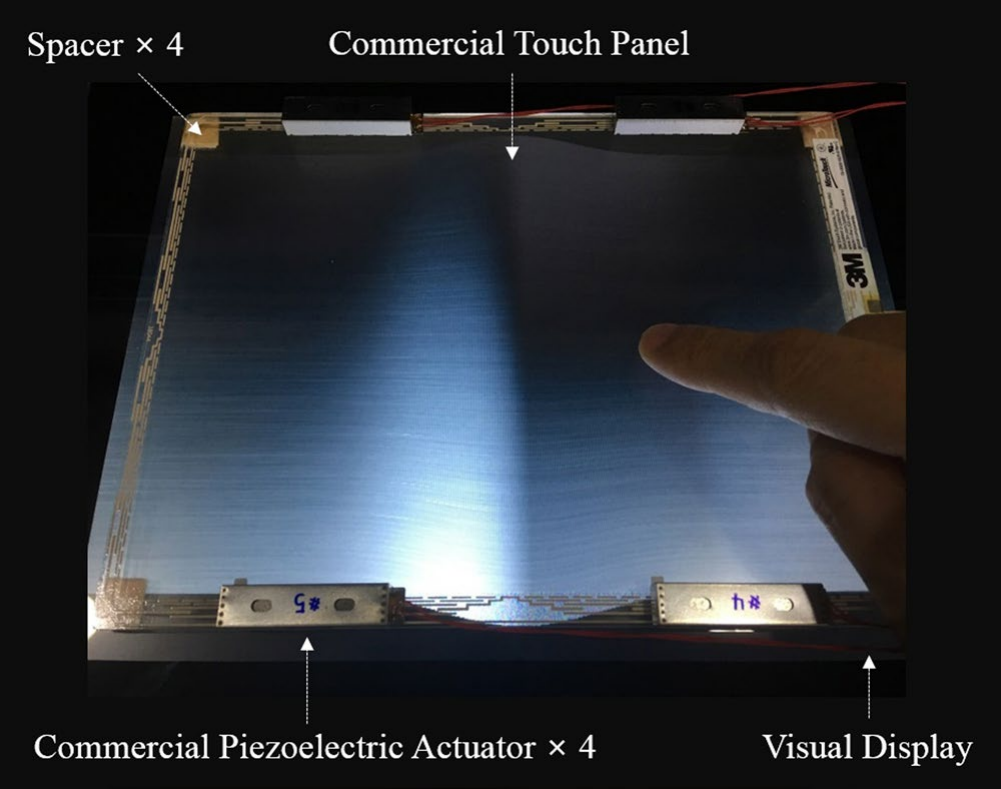
Hardware setup for generating both electrovibration and mechanical vibration stimuli.

Figure 3 shows the constructed hardware setup for providing both electrovibration and mechanical vibration to the fingertip of the participant. The setup mainly consisted of a touch panel (SCT3250, 3 M Inc.) for providing electrovibration feedback, piezoelectric actuators (PHA 379060, Mplus Inc., natural frequency of 165 Hz) for providing mechanical vibration feedback, and a visual display (STL-100T, StarTech) for visually providing the status of the experimental process to the participants. The dimensions of the touch panel and of the piezoelectric actuator were 194.32 × 146.05 × 3.18 mm and 37 × 9 × 6 mm, respectively. The touch panel was mounted on the visual display using four spacers (sponge, dimensions of 10 × 10 × 10 mm), which were placed at the edge of the touch panel. Then, four piezoelectric actuators were attached to the periphery of the touch panel. To produce electrovibration and mechanical vibration feedback, first, a host PC generated two digital signals. These signals were converted to analog signals via two signal processors (NI 9264, National Instruments Inc.). The analog signals were amplified by an analog amplifier (SQV 3/500, US Eurotek Inc.) and a piezoelectric driver (DRV8662, Texas Instruments Inc.), and were then applied to the touch panel and to the piezoelectric actuators in order to generate electrovibration and mechanical vibration, respectively. Four piezoelectric actuators were driven with identical frequency, phase, and amplitude. Electrovibration was generated by applying an alternating current (AC) signal to an electrode of the touch panel (see Supplementary Fig. S1). The method for producing mechanical vibration is shown in Supplementary Fig. S2. Four piezoelectric actuators, which were attached to the periphery of the touch panel, generated physical vibration in the vertical direction, in accordance with the applied electric signal. The vibrations that were produced by the piezoelectric actuators were transferred to the touch panel in a manner in which the entire surface could physically vibrate (see Supplementary Figs S3 through 6). Note that the physical interference between the electrovibration and the mechanical vibration was assumed to be negligible^13^.

### Stimuli

In Experiment 1, to investigate the absolute threshold of electrovibration, participants were exposed to both the test stimulus, *A*_*test*_, and the reference stimulus, *A*_*ref*_, during the sliding of their fingertip on the touch panel. In this experiment, *A*_*test*_ was the case where the electrovibration stimulus was provided, and *A*_*ref*_ was when the stimulus was not provided. A signal, in which a direct current (DC) offset had been added to the pure sine wave, was used to generate the electrovibration stimulus because when a person recognizes an electrovibration, he or she is provided with feedback that is proportional to the absolute value of the electric potential difference^42^. The signal for providing the test stimulus, *A*_*test*_, was generated in the form of *V*_*e*_(*t*) = *A*_*e*_sin(2*πf*_*e*_*t*) + *A*_*e*_, where *A*_*e*_ and *f*_*e*_ denote the amplitude and the frequency of the electrovibration, respectively. The frequencies of the test stimuli were 120, 180, and 270 Hz, and were arranged at regular intervals on a logarithmic scale. Accordingly, in the experiment for the investigation of the absolute threshold of mechanical vibration, participants were exposed to both the test stimulus and the reference stimulus during the sliding of their fingertip on the touch panel; the mechanical vibration was provided only in the test stimulus. The signal for generating the mechanical vibration stimulus was a pure sine wave, *V*_*m*_(*t*) = *A*_*m*_sin(2*πf*_*m*_*t*), where *A*_*m*_ and *f*_*m*_ denote the amplitude and the frequency of the mechanical vibration, respectively. Considering that the piezoelectric actuator that was used in the experiment vibrated vertically according to the sign of the applied voltage, the pure sine wave signal was applied in the case of mechanical vibration. Moreover, the frequencies of the mechanical-vibration test stimuli were set to 120, 180, and 270 Hz as well, and were equally spaced on the logarithmic scale. Each stimulus was provided for 1.8 s. Table 1 lists the test stimuli that were used in the experiments.

**Table 1:** Test stimuli set for Experiment 1: absolute threshold of electrovibration and mechanical vibration.

Feedback type	Signal applied to apparatus	Test frequency [Hz]
Electrovibration (EV)	Sine wave (DC offset), *V*_*e*_(*t*) = *A*_*e*_sin(2*πf*_*e*_*t*) + *A*_*e*_	120, 180, 270
Mechanical vibration (MV)	Pure sine wave, *V*_*m*_(*t*) = *A*_*m*_sin(2*πf*_*m*_*t*)	120, 180, 270

In Experiment 2, to investigate the absolute threshold of electrovibration in the presence of mechanical vibration, participants were exposed to both the test stimulus, *A*_*test*_, and reference stimulus, *A*_*ref*_, during the sliding of their fingertip on the touch panel. The electrovibration was provided only in the test stimulus, whereas the mechanical vibration was provided as the masking stimulus at the same frequency and intensity for both the test stimulus and the reference stimulus. Table 2 lists the test stimuli set that was used in Experiment 2. The waveform of the stimulus was the same as that in Experiment 1. The frequency of 270 Hz was selected for the electrovibration because participants were relatively sensitive to this frequency (results from Experiment 1) and because it was perceived to be closer to friction rather than to vibration^11^. The frequencies of the mechanical vibration were 120, 180, and 270 Hz, and intensities from 5 dB SL to 25 dB SL (increment of 5 dB SL) were used for each frequency. The signal amplitudes of the mechanical vibration in units of dB SL were derived from the results of Experiment 1. Each stimulus was provided for 1.8 s.

**Table 2:** Test stimuli set for Experiment 2: absolute threshold of electrovibration in the presence of mechanical vibration.

	Set 1–5	Set 6–10	Set 11–15
Frequency of EV (Hz)	270	270	270
Frequency of MV (Hz)	120	180	270
Intensity of MV (dB SL)	5, 10, 15, 20, 25	5, 10, 15, 20, 25	5, 10, 15, 20, 25

In Experiment 3, to investigate the difference threshold of electrovibration in the presence of mechanical vibration, participants were exposed to both the test stimulus, *A*_*test*_ = *A*_*ref*_ + Δ*A*, and reference stimulus, *A*_*ref*_, during the sliding of their fingertip on the touch panel, where Δ*A* is a variable amplitude increment. The values of *A*_*ref*_ were set to 15 dB SL for all test sets, according to the masked threshold that was investigated in Experiments 1 and 2. During an experimental run, mechanical vibration was provided as the masking stimulus at the same frequency and intensity for both the test stimulus and the reference stimulus. As in Experiment 2, the frequency of 270 Hz was selected for electrovibration; the frequencies and the intensity of the mechanical vibration were 120, 180, and 270 Hz, and 10 dB SL and 20 dB SL, respectively. In addition, the waveform of the stimulus was the same as that in Experiment 2. In addition, for comparison purposes, a test set was included in Experiment 3 to investigate the difference threshold when only electrovibration was provided (mechanical vibration was not provided in this case: Set 7 in Table 3).

**Table 3:** Test stimuli set for Experiment 3: difference threshold of electrovibration in the presence of mechanical vibration.

	Set 1	Set 2	Set 3	Set 4	Set 5	Set 6	Set 7
Frequency of EV (Hz)	270	270	270	270	270	270	270
Intensity of EV (dB)	*A*_*test*_ = *A*_*ref*_ + Δ*A*, where, *A*_*ref*_ : 15 dB SL, Δ*A*: variable increment (dB)						
Frequency of MV (Hz)	120	120	180	180	270	270	—
Intensity of MV (dB SL)	10	20	10	20	10	20	—

### Procedure

**Figure 4:**
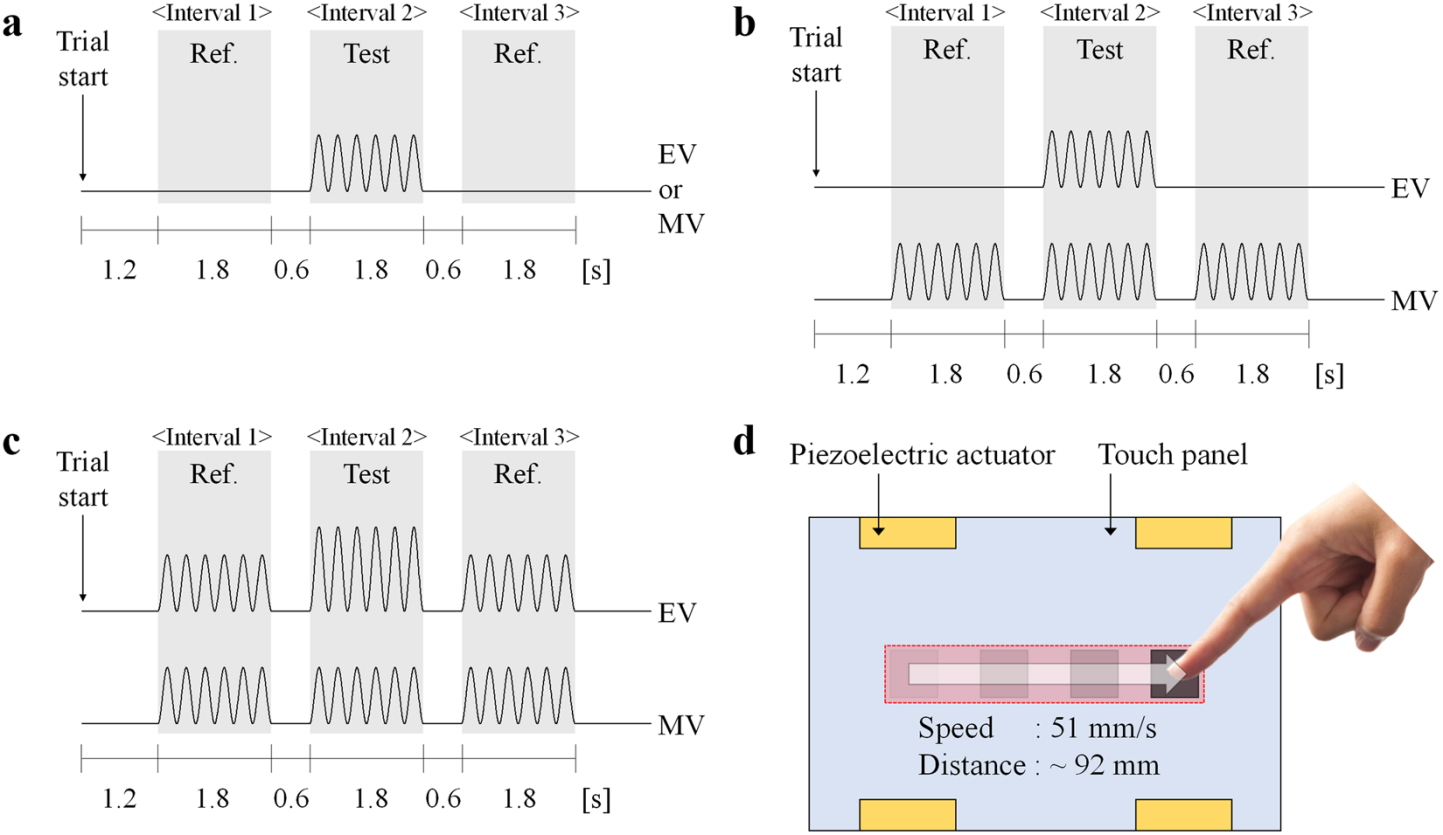
Stimulus sequence within a three-interval, forced-choice (3IFC) paradigm. (**a**) Stimulus sequence in Experiment 1. (**b**) Stimulus sequence in Experiment 2. (**c**) Stimulus sequence in Experiment 3. (**d**) Sliding speed and distance in each interval during the experimental run.

The same experimental procedure, namely a three-interval forced-choice (3IFC) paradigm with a one-up three-down adaptive procedure^56,57^, was used for all three experiments. In each trial, participants were given three stimulus presentations. The test stimulus, *A*_*test*_, was provided at one randomly selected interval out of the three intervals; the reference stimulus, *A*_*ref*_, was provided at the other two intervals. The total duration of each trial was 7.8 s. The first trial of each session allowed the participants to start by pressing the “spacebar” on the keyboard. After a 1.2 s wait, the first stimulus was provided for 1.8 s, and the next stimulus was provided sequentially, with an inter-stimulus interval of 0.6 s (See Fig. 4a through c). During each of the 1.8 s interval in which the test or the reference stimulus was provided, the participants were instructed to slide their fingertip from the left to the right along the graphic object, moving at a constant speed on the visual display, as shown in Fig. 4d. The dimensions of the graphic object were 13 × 13 mm. The sliding speed was fixed at 51 mm/s, and was determined by considering the speed range at which a person would typically move to recognize objects^13,58^. No specific restrictions were placed on other parameters (such as the contact force and the area), except for the sliding speed; each participant performed the experiment under his/her usual “natural touch” condition. However, participants were instructed to keep their touch conditions as constant as possible throughout the entire experiment. The task of each participant was to indicate the “odd” interval that contained the test stimulus by pressing “1”, “2”, or “3” on the keyboard. The next trial started immediately after the response was submitted. When it was difficult for participants to be confident about the interval in which the test stimulus was contained, they were instructed to speculate. They were unable to perform the trial repeatedly and were not allowed to return to the previous trial. In addition, no feedback on whether they submitted the correct answer was provided during the experiments.

For each session, the intensity of the stimulus in the first trial (i.e., the initial value of *A*_*test*_ for Expriments 1 and 2, or Δ*A* for Experiment 3) was made to be sufficiently higher than the expected absolute or difference threshold. If the participant submitted three consecutive correct responses, the value of *A*_*test*_ or Δ*A* in the next trial decreased. On the other hand, if the participant submitted a wrong answer once, the value of *A*_*test*_ or Δ*A* increased in the next trial. The point at which the value of *A*_*test*_ or Δ*A* changes from decreasing to increasing, or vice versa, is referred to as “reversal”. The value of *A*_*test*_ or Δ*A* initially changed by 2 dB for faster convergence, and then by 0.5 dB after the first three reversals for better accuracy in the threshold estimate. Each session was terminated after eight reversals of a 0.5 dB step size. Each session typically required 60–80 trials and lasted for approximately 9–15 min. Each time the session ended, the participant had to rest for more than 5 min. Experiment 1 consisted of six sessions (2 feedback types × 3 test frequencies per each feedback type); the order of sessions was random for each participant. The Experiment 2 consisted of fifteen sessions (3 frequencies of the masking stimulus × 5 amplitudes per frequency). Again, the order of sessions was random for each participant. Assuming that a typical session was completed after 70 trials, each participant performed a total of 420 trials and 1050 trials and spent approximately 90 min and 3 hours, including enough breaks, for Experiments 1 and 2, respectively. Experiment 3 consisted of seven sessions (3 frequencies of the masking stimulus × 2 amplitudes per frequency + no-masker condition); the order of sessions was random for each participant. Each participant performed approximately 490 trials for Experiment 3, which lasted for approximately 90 min, including breaks. Each of the three experiments was performed at intervals of at least one day.

During the experiments, all participants wore an adjustable wrist band with a 1 MΩ resistor (ECWS61M-1, 3 M Inc.) on their wrist of the dominant hand, which was used for grounding. Headphones and pink noise (presented roughly at 80 dB SPL) were used for the prevention of possible auditory interference. A small amount of talcum powder was allowed to be used before starting the experiment to prevent the increase in friction due to sweat. A training session was provided prior to the start of each experiment. Participants were instructed to practice on various test sets to adapt to the experimental procedures and to be able to make the distinction between the test stimulus and the reference stimulus. Participants were able to terminate training at any time when they deemed themselves ready; the training session typically lasted for less than a few minutes. Immediately after the training session, the main experiment was conducted, along with data collection.

### Data analysis

Out of the four pairs of *A*_*test*_ or Δ*A* values that were obtained from the last eight reversals in a small step size, four threshold estimates were obtained by averaging the peak–valley pairs. The absolute or difference threshold, in decibels, was then estimated by averaging the four threshold estimates. The estimated threshold corresponds to the 79.4-percentile point on the psychometric function^56^. To evaluate the masking effects, the ALs or DLs of EV in no-masker conditions were subtracted from the corresponding masked ALs or DLs; these changes in ALs or DLs were used for analysis. Prior to the main analysis, Shapiro–Wilk test was performed to confirm if the normality assumption was met. All experimental data were then analyzed via repeated-measures ANOVA. Depending on the sphericity that was obtained from Mauchly’s test, a correction was accordingly applied. A one-sample *t*-test was used when a test was needed in order to assess whether the data set differed from zero. For all analyses, *p* < 0.05 was deemed significant.

### Data availability statement

The data obtained are available as supplementary information.

## Electronic supplementary material

Supplementary Information
