# Supplementary material for: Mechanical Vibration Influences the Perception of Electrovibration
Source: Sci Rep. 2018 Mar 14;8:4555. doi: 10.1038/s41598-018-22865-x (PMC5852143; doi:10.1038/s41598-018-22865-x)
Supplement: Supplementary file 1 — Supplementary Information [file 41598_2018_22865_MOESM1_ESM.pdf]

Mechanical Vibration Influences the Perception of Electrovibration

Semin Ryu[1], Dongbum Pyo[2], Soo-Chul Lim[3], and Dong-Soo Kwon[4,*]

[1] Department of Interdisciplinary Studies, Hallym University, 1 Hallymdaehak-gil, Chuncheon-si, Gangwon-do 24252, Republic of Korea
[2] Electronics and Telecommunications Research Institute (ETRI), 218 Gajeong-ro, Yuseong-gu, Daejeon 34129, Republic of Korea
[3] Department of Mechanical, Robotics and Energy Engineering, Dongguk University-Seoul, 30 Pildong-ro 1-gil, Jung-gu, Seoul 04620, Republic of Korea
[4] Department of Mechanical Engineering, Korea Advanced Institute of Science and Technology (KAIST), 291 Daehak-ro, Yuseong-gu, Daejeon 34141, Republic of Korea

[*] Corresponding author. Phone: +82 42 350 3042, Fax: +82 42 350 8240, E-mail address: kwonds@kaist.ac.kr

Supplementary Information

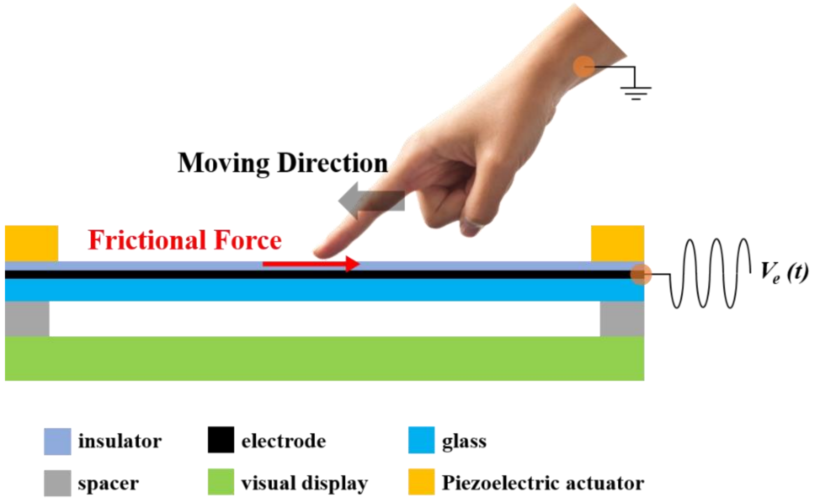

Figure S1. Generation of electrovibration. The touch panel mainly consists of glass, an electrode, and an insulating layer. When an alternating current (AC) signal is applied to an electrode, an electrostatic attractive force is periodically generated between the skin of the fingertip and the electrode. These forces are not perceived when the fingertip is immobile; however, they act in the form of frictional forces that can only be perceived while the fingertip is sliding. The perceived intensity of the frictional force can be modulated by varying the frequency and the amplitude of the applied signal.

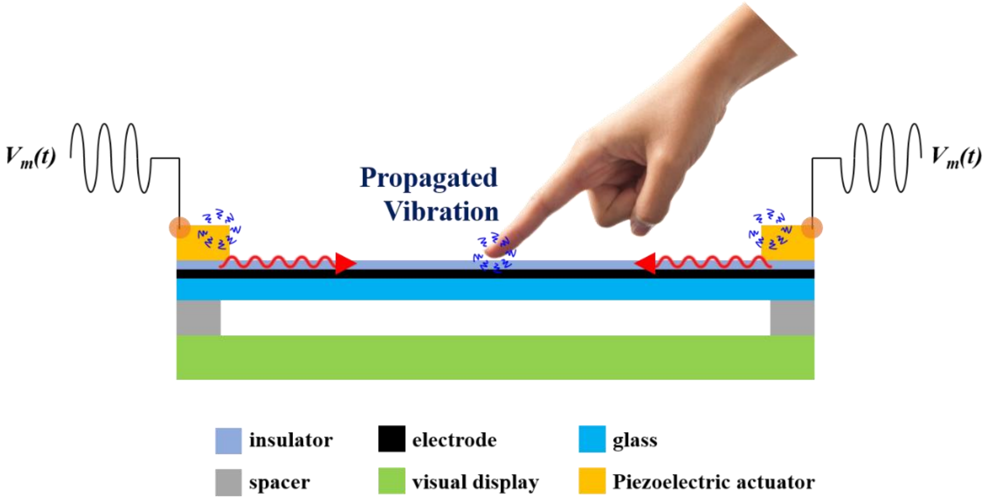

Figure S2. Generation of mechanical vibration. The four piezoelectric actuators that are attached to the periphery of the touch panel generate mechanical vibration in the vertical direction by the applied AC signal. The vibration that is generated by the piezoelectric actuators propagates on the touch panel, causing the entire surface to vibrate vertically. That is, a vibration occurs on the entire surface, in a direction perpendicular to the direction in which the fingertip slides.

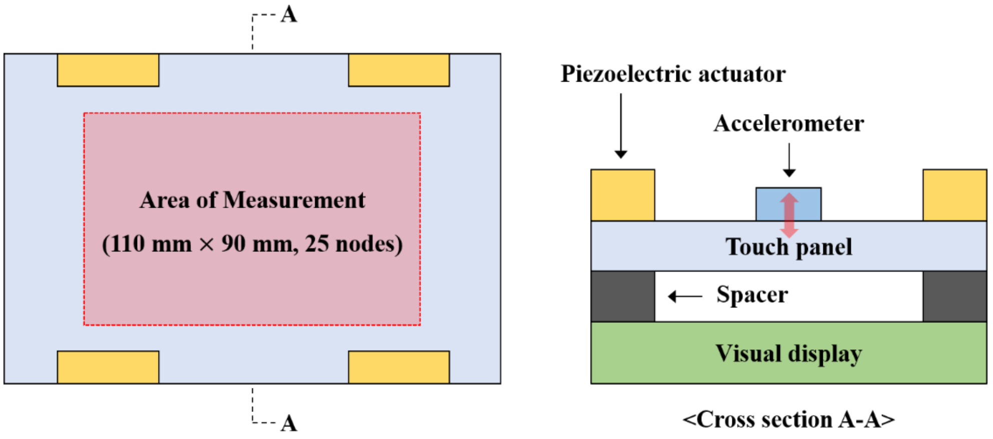

Figure S3. Experimental setup for measuring the acceleration of the touch surface. To measure the vertical acceleration of the vibration that is transmitted to the touch panel surface, the measurement setup with an accelerometer (ADXL203, Analog Devices) was constructed. The mass of the sensor was less than approximately 1/100 of the mass of the touch panel; thus, the influence of the accelerometer on system characteristics was assumed negligible. The measurements were performed on 25 nodes, which were arranged at equal intervals within a specific area (100 mm × 90 mm) on the touch panel.

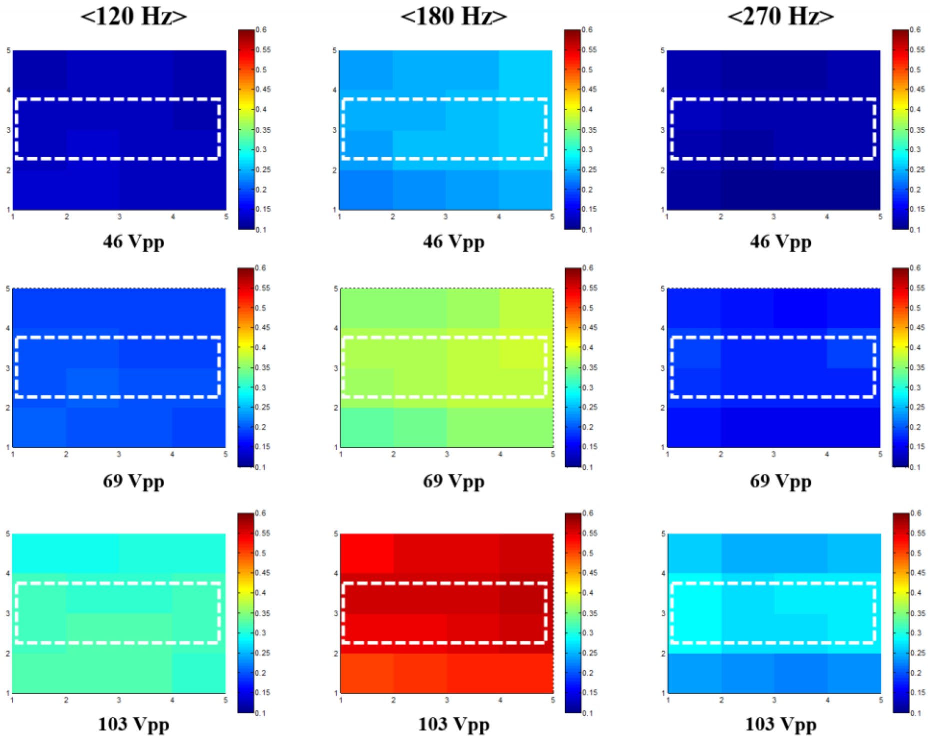

Figure S4. Measured acceleration at 25 nodes on the target area for different conditions of frequency and amplitude. The acceleration was measured in units of G (standard gravity) at 25 nodes for several frequencies and amplitudes of the input signal. The maximum value of the acceleration deviation within 25 nodes for all measurement conditions was approximately 20 %. However, the acceleration deviations in the certain area (enclosed within the white dashed box), except for the 10 nodes adjacent to the piezoelectric actuators, were found to be within approximately 2 to 10 % for all measurement conditions. The deviation of the acceleration would not be perceived when sliding a fingertip within the corresponding area, considering that the difference threshold for the intensity of the vibration has been known to be approximately 15–40 %. Hence, in this work, the experiments were conducted by sliding the fingertip in the area enclosed within the white dashed box.

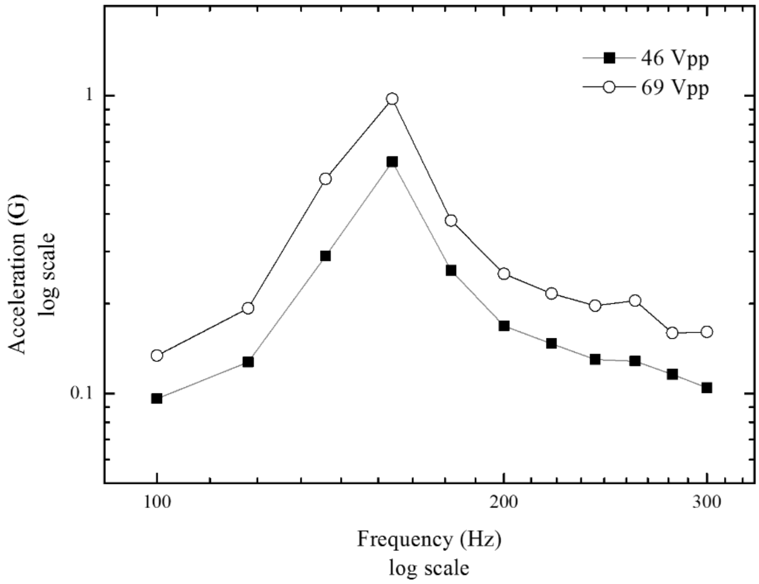

Figure S5. Measured acceleration according to the frequency of the input signal for different voltages. The acceleration was measured at the center of the touch panel in units of G (standard gravity) as a function of the frequency of the input signal. The acceleration of the surface of the touch panel presented similar characteristics depending on the frequency, regardless of the intensity of the applied signal. The acceleration gradually increases with the increase in frequency, it reaches a maximum value at approximately 160 Hz, and then gradually decreases. The expected resonant frequency of 160 Hz corresponds to the resonance of the piezoelectric actuator.

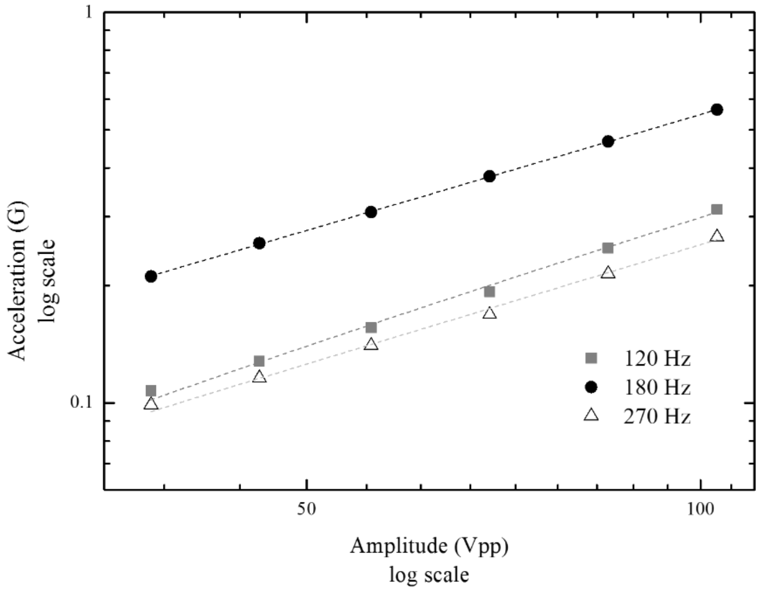

Figure S6. Measured acceleration according to the voltage of the input signal for different frequencies. The acceleration was measured at the center of the touch panel in units of G (standard gravity) as a function of the intensity of the input signal. The acceleration of the surface of the touch panel tends to increase with the increase in the applied voltage, regardless of the frequency. The dotted straight line denotes a linear regression of the measured values for each frequency. The relationship between the applied voltage and the acceleration is linear on the log–log scale (R-square > 0.99 for the three test frequencies).

Table S1. Results of Experiment 1. Absolute threshold (AL) of each of electrovibration (EV) and mechanical vibration (MV). The values imply mean ± standard error.

	Set 1	Set 2	Set 3	Set 4	Set 5	Set 6
**Feedback type**	Electrovibration (EV)			Mechanical vibration (MV)		
**Frequency (Hz)**	120	180	270	120	180	270
**Absolute threshold (dB re Vpp for EV, m/s^2 for MV)**	20.760 ± 1.017	16.726 ± 0.778	18.029 ± 1.429	0.080 ± 0.009	0.123 ± 0.017	0.207 ± 0.022

Table S2. Results of Experiment 2. Changes in absolute threshold (AL) of electrovibration (EV) in the presence of masking stimuli, mechanical vibration (MV). The values imply mean ± standard error.

	Set 1	Set 2	Set 3	Set 4	Set 5
**Frequency of EV (Hz)**			270		
**Frequency of MV (Hz)**			120		
**Intensity of MV (dB SL)**	5	10	15	20	25
**Changes in AL of EV (dB)**	0.433 ± 0.383	0.322 ± 0.365	2.992 ± 1.099	7.265 ± 0.777	10.107 ± 0.714

	Set 6	Set 7	Set 8	Set 9	Set 10
**Frequency of EV (Hz)**			270		
**Frequency of MV (Hz)**			180		
**Intensity of MV (dB SL)**	5	10	15	20	25
**Changes in AL of EV (dB)**	0.771 ± 0.393	0.808 ± 0.347	3.611 ± 0.615	7.427 ± 0.789	10.502 ± 1.150

	Set 11	Set 12	Set 13	Set 14	Set 15
**Frequency of EV (Hz)**			270		
**Frequency of MV (Hz)**			270		
**Intensity of MV (dB SL)**	5	10	15	20	25
**Changes in AL of EV (dB)**	1.171 ± 0.435	3.608 ± 0.602	6.411 ± 1.056	9.428 ± 0.649	13.301 ± 1.199

Table S3. Results of Experiment 3. Changes in difference threshold (DL) of electrovibration (EV) in the presence of masking stimuli, mechanical vibration (MV). The values imply mean ± standard error.

	Set 1	Set 2	Set 3	Set 4	Set 5	Set 6
**Frequency of EV (Hz)**			270			
**Frequency of MV (Hz)**	120	120	180	180	270	270
**Intensity of MV (dB SL)**	10	20	10	20	10	20
**Changes in DL of EV (dB)**	0.210 ± 0.147	0.256 ± 0.137	0.201 ± 0.158	0.236 ± 0.215	0.202 ± 0.230	0.393 ± 0.176
